# Oncological Care During a Crisis: A Systematic Review and Meta-Analysis of Non-Melanoma Skin Cancer of the Head and Neck Region in the COVID-19 Pandemic

**DOI:** 10.3390/diseases14070254

**Published:** 2026-07-14

**Authors:** Andrea Frosolini, Simone Benedetti, Luigi Angelo Vaira, Guido Gabriele, Paolo Gennaro

**Affiliations:** 1 Maxillofacial Surgery Unit, Department of Medical Biotechnology, S. Maria Alle Scotte University Hospital of Siena, 53100 Siena, Italy; simone.benedetti1992@gmail.com (S.B.); guido.gabriele@unisi.it (G.G.); paolo.gennaro@unisi.it (P.G.); 2 Maxillofacial Surgery Operative Unit, Department of Medicine, Surgery and Pharmacy, University of Sassari, 07100 Sassari, Italy; lavaira@uniss.it

**Keywords:** COVID-19, non-melanoma skin cancer (NMSC), head and neck surgery, pandemic healthcare, reconstructive surgery

## Abstract

Introduction: The COVID-19 pandemic profoundly disrupted healthcare, prompting adaptations in oncological practices. This systematic review evaluates the pandemic’s impact on the management of non-melanoma skin cancer (NMSC) of the head and neck (H&N) region. Methods: The review was reported according to PRISMA guidelines. Eligible studies compared pre-pandemic and pandemic periods in adult patients treated for head and neck NMSC reporting outcomes related to histological distribution, time to treatment initiation, reconstructive method, and surgical margin status. Risk of bias was assessed using the Newcastle–Ottawa Scale. Random-effects meta-analyses were performed for outcomes with sufficient extractable data. Results: The review comprised five studies involving 1318 cases. The relative distribution of basal cell carcinoma and squamous cell carcinoma did not differ significantly between pre-pandemic and pandemic periods. Time to treatment initiation showed no significant overall change, although heterogeneity was high, indicating substantial variability among healthcare settings. Reconstruction shifted significantly toward primary closure compared with flap reconstruction during the pandemic period (OR 1.90; 95% CI: 1.46–2.47; *p* < 0.0001). Negative surgical margins were reported in 1119 of 1266 excisions/cases with available data (88.4%), with no significant difference between pre-pandemic and pandemic periods (OR 0.77; 95% CI: 0.46–1.32; *p* = 0.34). Conclusions: The pandemic was associated with a shift toward simpler reconstructive strategies, particularly increased use of primary closure, likely reflecting attempts to reduce operative complexity and resource use. While short-term oncologic outcomes appeared broadly preserved, long-term functional, aesthetic, and patient-reported outcomes remain insufficiently characterized. Future studies should evaluate these outcomes to better inform crisis-adapted oncologic and reconstructive care pathways.

## 1. Introduction

Non-Melanoma Skin Cancer (NMSC), primarily Basal Cell Carcinoma (BCC) and Squamous Cell Carcinoma (SCC), is the most common type of skin cancer [1] and is largely linked to high ultraviolet exposure [2]. Over 85% of cases affect the head and neck, often requiring maxillofacial surgeons for radical treatment, functional preservation, and aesthetic reconstruction [3,4].

The onset of the coronavirus disease 2019 (COVID-19) pandemic, caused by severe acute respiratory syndrome coronavirus 2 (SARS-CoV-2), disrupted healthcare systems worldwide. Since its emergence in early 2020, the pandemic has forced a reallocation of healthcare resources, prioritizing COVID-19 patient management [5,6]. Despite efforts to maintain care levels, many specialties experienced delays in diagnosis and treatment due to overwhelmed hospital systems, COVID-19 precautions, and workforce reallocation. Some studies reported surgical de-escalation for cancer treatments in favor of nonsurgical approaches, particularly during the pandemic’s early stages [7]. Others noted a significant decline in cancer screening and treatment, suggesting a delayed impact of the pandemic on cancer care [8,9]. A recent study in the USA highlighted a significant reduction in new cancer diagnoses and early-stage cancers during 2020, highlighting the need for evaluation of the pandemic’s long-term effects on cancer morbidity and mortality [10]. For NMSC, the pandemic’s impact has been inconsistent.

While some studies reported significant treatment delays potentially leading to more advanced or aggressive NMSC presentations [11,12], others found no substantial changes in diagnosis or management patterns [13,14]. During the same period, the therapeutic landscape of advanced NMSC was also evolving, particularly with the increasing use of immuno-oncology treatments, such as anti–PD-1 immune checkpoint inhibitors, for selected patients with locally advanced or unresectable cutaneous squamous cell carcinoma [15]. This may have influenced patient selection for surgery in some settings, although surgical studies rarely reported treatment allocation according to systemic therapy eligibility. This inconsistency among single-center experiences highlights the need for a systematic synthesis of the available evidence. Although the acute phase of the COVID-19 pandemic has ended, its consequences for oncologic surgical pathways remain clinically relevant. The pandemic represented a real-world stress test for healthcare systems, with widespread effects on surgical timing, resource allocation, operating-room access, and prioritization of oncologic procedures. This issue is particularly important for NMSC of the head and neck (H&N) region because surgical success is not determined only by oncologic clearance, but also by reconstructive quality, functional restoration, aesthetic outcomes, and patient-reported quality of life [3]. Understanding whether pandemic-related adaptations affected treatment timing, margin status, or reconstructive choices may therefore inform post-pandemic recovery strategies and contingency planning for future healthcare crises [4]. Therefore, this systematic review and meta-analysis aimed to investigate the impact of the COVID-19 pandemic on the oncologic and reconstructive management of H&N NMSC, with specific focus on histopathological trends, time to treatment, methods of reconstruction, and surgical margin status.

## 2. Materials and Methods

### 2.1. Study Protocol and Search Strategy

The protocol of the Systematic Review was registered on the PROSPERO database International prospective register of systematic reviews (Center for Reviews and Dissemination, University of York, York, UK), with ID number CRD42024596143. The review was reported in accordance with Preferred Reporting Items for Systematic Reviews and Meta-Analyses (PRISMA) guidelines. We performed a comprehensive literature search across three major databases: PubMed, Google Scholar, and Scopus. Significant search terms included “COVID-19,” “SARS-CoV-2,” “pandemic,” “non-melanoma skin cancer,” “Basal cell carcinoma,” “Squamous cell carcinoma,” “head and neck,” “diagnosis,” “management,” “treatment,” and “surgery.” These terms were combined using Boolean operators and tailored according to each database’s search interface (see Supplementary Materials). The literature search was performed on 4 May 2026.

### 2.2. Inclusion and Exclusion Criteria

We applied the following PICOS (Population, Intervention, Comparison, Outcome, and Study Design) criteria: (i) Studies involving patients with NMSC, including BCC and SCC, specifically located in the H&N region; (ii) Studies reporting on the diagnosis, management, treatment, or surgical intervention for NMSC during the COVID-19 pandemic; (iii) Studies comparing the period before the pandemic (pre-2020) and during the pandemic (2020 onwards) in terms of diagnosis or management outcomes; (iv) as primary outcome, the changes in diagnosis rates, treatment delays, and surgical management of NMSC. As secondary outcome, healthcare resource allocation, treatment prioritization, and outcomes related to delayed care; (v) We included prospective and retrospective cohort studies, case–control studies, and cross-sectional studies. Additional inclusion criteria: (i) Articles available in English; (ii) Studies involving adult patients (18 years and older). We considered the following exclusion criteria: (i) Studies focusing solely on melanoma or other skin conditions; (ii) studies not providing clear differentiation between pre-pandemic and pandemic data; (iii) Non-human studies or those without sufficient statistical data; (iv) Reviews, case reports, editorials, and non-original research.

### 2.3. Data Extraction and Synthesis

Two independent reviewers (A.F., S.B.) screened the titles and abstracts of the retrieved records. Full-text articles were then assessed independently and in duplicate against the predefined inclusion/exclusion criteria. Inter-rater agreement was calculated using Cohen’s kappa, agreement for full-text eligibility assessment was complete, corresponding to Cohen’s κ = 1.00. Disagreements, when present, were resolved through discussion or by consulting a third researcher (G.G.).

Data extraction was performed using a standardized data-abstraction form developed for this review (see Supplementary Material). Data extraction was performed by one reviewer (A.F.) and checked by a second reviewer (S.B.) for accuracy and consistency. Any discrepancies were resolved by consensus. The primary unit of analysis was the patient when demographic or epidemiological outcomes were reported. For lesion- or procedure-related outcomes, including surgical margins and reconstructive methods, the unit of analysis followed the denominator reported in the original studies, usually lesions, excisions, or surgical cases. This distinction was preserved during data extraction and synthesis. The results from the selected studies were summarized through narrative synthesis. Outcomes with sufficiently comparable extractable data across studies were additionally pooled using meta-analysis.

### 2.4. Risk of Bias Assessment

The risk of bias for individual studies was assessed using the Newcastle–Ottawa Scale (NOS), since all included studies were observational studies [16]. Studies were categorized as low, moderate, or high risk of bias based on their methodological quality and potential confounders (a domain-level NOS assessment was also reported in Supplementary Table S1).

### 2.5. Statistical Analysis

For the meta-analysis, pooled effect sizes were calculated using a random-effects model [17], which was selected a priori because clinical and methodological heterogeneity was expected across studies, countries, healthcare systems, study periods, and local pandemic responses. Studies were pooled by outcome rather than by study design, and quantitative synthesis was performed only when outcome definitions and extractable data were sufficiently comparable across studies. Heterogeneity across studies was evaluated using the I^2^ statistic. Given the limited number of included studies, formal sensitivity analyses and publication-bias assessment using funnel plots or Egger’s test were not performed. To estimate any missing standard deviations, a weighted pooled standard deviation method was used, enabling consistent comparisons across studies. For continuous outcomes, time to treatment initiation was pooled using standardized mean differences (SMDs); positive SMD values indicated longer time to treatment initiation before the pandemic, whereas negative values indicated longer time to treatment initiation during the pandemic. To assess changes in key outcomes, odds ratios (ORs) were calculated for binary categorical outcomes, including histological distribution, surgical margin status, and reconstruction methods. The pandemic period was considered the exposure condition, while the pre-pandemic period served as the comparator, reflecting the before–after comparative design of the included studies. Each outcome was analyzed using a 2 × 2 contingency table structure to compare groups: outcomes were categorized by type (e.g., BCC vs. SCC, negative vs. positive margins, primary vs. flap reconstruction) and by period (pre-pandemic or pandemic). The odds of each outcome within each period were calculated, followed by the Odds Ratio (OR) to represent the relative change between periods: OR = (Outcome in Pandemic/Comparator in Pandemic)/(Outcome in Pre-Pandemic/Comparator in Pre-Pandemic). To evaluate the variability and precision of each OR, we applied natural logarithmic transformation before pooling, because log-transformed ORs are approximately normally distributed and allow standard error estimation and inverse-variance weighting [18]. Pooled estimates were subsequently back-transformed and reported as ORs with 95% confidence intervals (CIs). Statistical significance was set at *p* < 0.05. Meta-analysis was conducted using MetaAnalysisOnline.com, a web-based tool for rapid meta-analysis of clinical and epidemiological studies [19].

## 3. Results

### 3.1. General Characteristics and Risk of Bias Assessment of Included Studies

**Figure 1:**
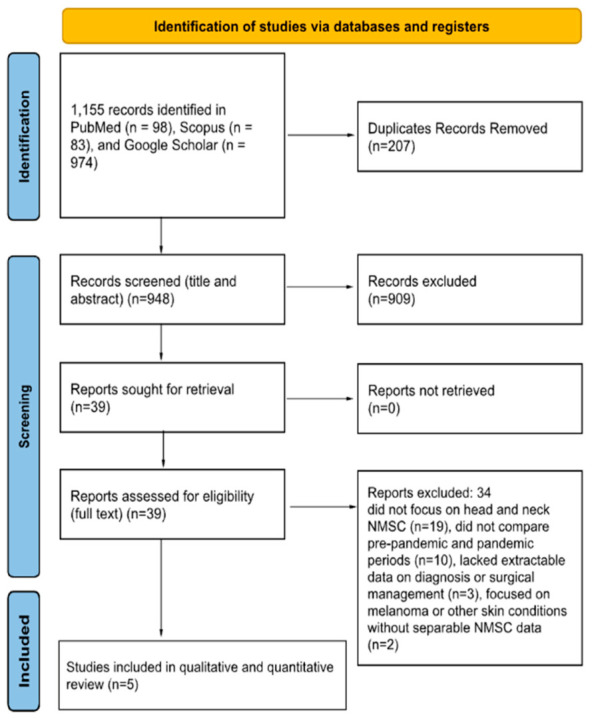
PRISMA literature search diagram from identification to inclusion.

The search identified 1155 records through database searches: PubMed (n = 98), Scopus (n = 83), and Google Scholar (n = 974). The selection process led to the final inclusion of five studies [11,13,14,20,21], as outlined in the PRISMA flow diagram, which also includes reasons for exclusion at the full-text stage (Figure 1). The included studies span different geographic regions and healthcare systems. Moreover, different pre-pandemic and pandemic periods were considered to assess changes in variables and outcomes. The NOS risk of bias assessment revealed three studies demonstrating low risk of bias [13,14,21] and two studies showing a moderate risk of bias [11,20]. The studies with low risk of bias were characterized by robust cohort selection, comparability of confounders (such as age, comorbidities, and tumor type), and adequate follow-up durations [13,14,21]. On the other hand, Seretis et al. [11] and Cozzi et al. [20] had a moderate risk of bias, primarily due to limited long-term follow-up and outcome reporting. Table 1 summarizes general characteristics and risk of bias assessment of included studies.

### 3.2. Epidemiological Trend, Diagnosis and Staging

The studies included in this review provide data on a combined total of 737 patients treated [11,14,20,21] accounting for 1318 head and neck skin cancer cases [11,13,14,20,21] across both pre-pandemic and pandemic periods (Table 2).

There was a predominance of male patients, with 456 males and 281 females out of 737 total patients [11,14,20,21], corresponding to approximately 61.9% male. Across the studies, Stage T1 was the most frequently recorded, with 385 cases out of 594 staged cases (64.8% of the total), followed by 141 cases out of 594 in Stage T2 (23.7%), 63 cases out of 594 in Stage T3 (10.6%), and 5 cases out of 594 in Stage T4 (0.8%), indicating that most reported lesions were early-stage tumors. BCC was consistently more common than SCC, with a total of 778 BCC cases out of 1318 reported histological diagnoses (59.0%) and 472 SCC cases (35.8%). Additionally, other histologies were minimally represented, accounting for 68 cases out of 1318 (5.2%), including malignant melanoma and pleomorphic sarcoma in studies that included broader skin cancer cohorts [11,13]. Tumor diameter, depth of invasion, and grading were reported across some studies with varying completeness. Tumor diameter was recorded in three studies, with values ranging from 13.3 ± 8.615 to 23.6 ± 17 mm, being 17.9 ± 14.5 the intermediate value. DOI was noted only in the Benedetti study, with an average of 4.59 ± 4 mm overall. Grading (G) was inconsistently reported with two studies documenting that G2 tumors constituted the largest group, accounting for 54 cases out of 99. G1 tumors were recorded in 36 cases out of 99, while Grade 3 and 4 cases were minimal with only 7 and 2 recorded instances across studies, respectively.

### 3.3. Management and Outcome

Local anesthesia (LA) was the preferred approach, with 446 cases out of 542 (82.3%) overall receiving LA and 96 cases out of 542 (17.7%) under general anesthesia (GA). Across the studies, 310 cases out of 901 (34.4%) were managed with primary repair, 413 cases out of 901 (45.8%) with flap reconstruction, and 93 cases out of 901 (10.3%) required skin grafts. Matrix reconstruction was less commonly utilized, with 85 cases out of 901 (9.4%) 11,13,14,16. Negative surgical margins were achieved in 1119 cases out of 1266 (88.4%), while 147 cases out of 1266 (11.6%) reported positive margins, indicating residual tumor presence [11,13,14,15,16]. Relative values for pre-pandemic and pandemic periods are reported in Table 3.

### 3.4. Pre-Pandemic vs. Pandemic Meta-Analysis

**Figure 2:**
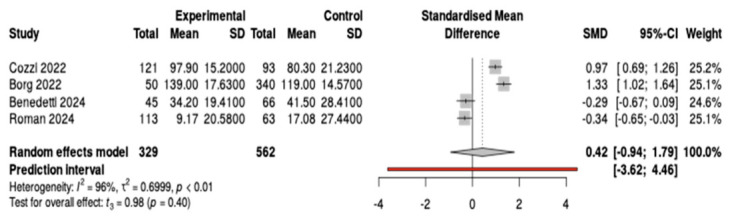
Time to treatment initiation (TTI) before and during COVID-19. Forest plot of TTI; positive SMDs indicate longer TTI before the pandemic, while negative SMDs indicate longer TTI during the pandemic.

The meta-analysis revealed differing impacts of the pandemic on various aspects of skin cancer treatment. The analysis of TTI showed no significant overall effect (t = 0.98; *p* = 0.40), suggesting that the pandemic did not universally increase or decrease TTI across the studies. The SMD was 0.42, with a 95% confidence interval of [−0.94; 1.79], reflecting a wide range of effects. Importantly, heterogeneity was high (I^2^ = 96%; *p* < 0.01), revealing substantial variability in TTI changes across studies, likely due to differing local pandemic conditions and healthcare responses (see Figure 2).

**Figure 3:**
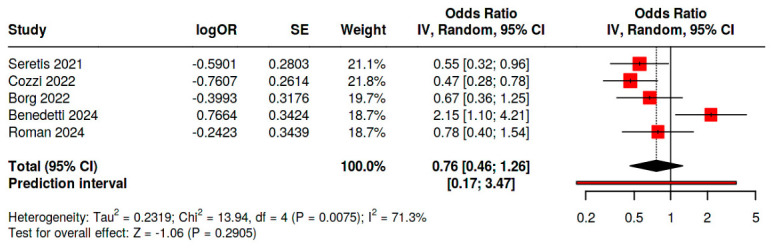
Histological distribution of BCC and SCC before and during the COVID-19 pandemic. Forest plot comparing BCC versus SCC. OR > 1 indicates a relative increase in BCC during the pandemic, whereas OR < 1 indicates a relative increase in SCC.

For histological distribution between BCC and SCC, no statistically significant change was observed between pre-pandemic and pandemic periods (OR: 0.76; 95% CI: 0.46–1.26; Z = −1.06; *p* = 0.2905). Heterogeneity was substantial (I^2^ = 71.3%; *p* = 0.0075), indicating variability among study-level estimates and suggesting that histological case mix may have differed across settings rather than showing a uniform pandemic-related pattern (see Figure 3).

**Figure 4:**
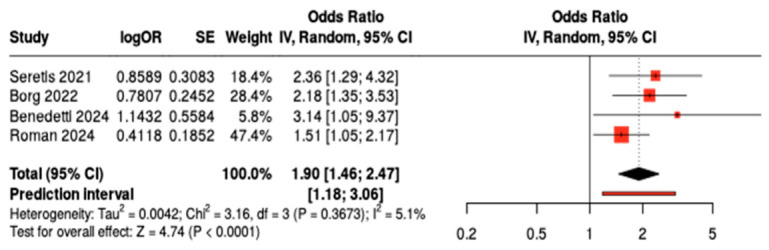
Reconstructive strategy before and during the COVID-19 pandemic. Forest plot comparing primary closure versus flap reconstruction. OR > 1 indicates a relative increase in primary closure during the pandemic; OR < 1 indicates a relative increase in flap reconstruction.

For reconstruction methods, a significant overall effect was observed, indicating a shift toward primary closure compared with flap reconstruction during the pandemic period (Z = 4.74; *p* < 0.0001). The pooled OR for primary closure versus flap reconstruction was 1.90 (95% CI: 1.46–2.47), suggesting a significantly higher relative use of primary closure during the pandemic. Heterogeneity was low (I^2^ = 5.1%; τ^2^ = 0.0042; χ^2^ = 3.16, df = 3, *p* = 0.3673), indicating consistency across studies in the observed reconstructive shift (Figure 4).

**Figure 5:**
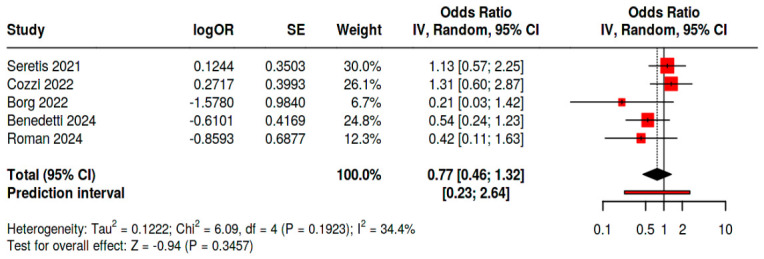
Surgical margin status before and during the COVID-19 pandemic. Forest plot comparing negative versus positive margins. OR > 1 indicates a relative increase in negative margins during the pandemic; OR < 1 indicates a relative increase in positive margins.

For surgical margin status, no statistically significant difference was observed between the pre-pandemic and pandemic periods. The pooled OR for negative versus positive margins was 0.77 (95% CI: 0.46–1.32; Z = −0.94; *p* = 0.3457), indicating no clear pandemic-related change in the likelihood of achieving negative margins. Heterogeneity was low to moderate (I^2^ = 34.4%; *p* = 0.1923), suggesting some variability among studies but no substantial inconsistency (see Figure 5).

## 4. Discussion

The COVID-19 pandemic caused widespread reorganization of healthcare services, with significant consequences for oncologic surgery due to resource reallocation, staff shortages, and reduced operating room availability [22,23,24,25]. Several centers reported delays in cancer diagnosis and treatment, as well as a temporary shift toward shorter and less resource-intensive procedures to limit hospitalization and viral exposure risks [26].

This review is one of the first systematic reviews with exploratory meta-analysis examining the pandemic’s impact on NMSC of the H&N region. The head and neck region poses unique challenges for NMSC treatment due to the high potential for functional and aesthetic impact, necessitating careful balance between radical treatment and preservation of form and function [27]. This review, aggregating findings from diverse geographic regions and healthcare systems, indicates that the pandemic had a limited measurable impact on short-term oncologic indicators, particularly the histological case mix and surgical margin status. However, this should not be interpreted as an absence of impact on care delivery. The stability in the relative proportions of BCC and SCC may reflect the slow-growing nature of most NMSC cases, which may not require urgent intervention compared to other rapidly progressing malignancies. Nevertheless, some studies reported increased aggressiveness in NMSC cases diagnosed during the pandemic, potentially due to delayed diagnoses [11]. Given the predominantly early-stage nature of cases included in this analysis, it appears that, while delays occurred, they may not have universally resulted in significant upstaging.

### 4.1. Reconstructive Strategy and Outcomes

A key finding of this review is the shift in reconstructive strategies during the pandemic, with an increased preference for primary repairs over more complex flap reconstructions. This shift likely reflects an adaptation in surgical strategy to minimize operating room time, reduce anesthesia-related risks, and conserve healthcare resources amid heightened pressures on hospitals. Primary closures are generally quicker to perform, involve fewer intraoperative steps, and require less surgical and post-operative care compared to more complex flap reconstructions. As such, the increased use of primary closure during the pandemic aligns with broader goals to reduce operative complexity, hospital exposure, and resource use, thereby limiting COVID-19 exposure risks for both patients and healthcare staff. However, this preference for primary closure over flap reconstruction comes with potential drawbacks, particularly regarding functional and aesthetic outcomes [3]. In head and neck NMSC, complex reconstructions, like local flaps, often play a critical role in preserving facial symmetry, function, and skin texture in sensitive areas. By contrast, primary closures, while efficient, may lead to suboptimal outcomes in cases where tension lines, skin laxity, or defect size are less favorable [28,29]. This can result in higher risks of scarring, contour irregularities, or functional impairment, particularly in areas around the eyes, nose, and mouth [30]. The pandemic-driven shift toward primary closure highlights the importance of carefully balancing short-term procedural simplification with long-term outcomes. As healthcare systems plan for future crises, it may be beneficial to refine protocols that prioritize more resource-efficient techniques without compromising the quality of reconstructive outcomes, where possible. Future studies should evaluate the post-pandemic impact of these choices on patient satisfaction and functional results, providing a basis for crisis-adapted guidelines in oncologic reconstructive surgery. Time to treatment initiation did not show a statistically significant pooled change across studies; however, this finding should be interpreted cautiously because heterogeneity was substantial. This finding is particularly noteworthy, as many healthcare resources were redirected toward managing COVID-19 patients. For many NMSC cases, especially lower-risk tumors, short treatment delays may have been less clinically consequential than for rapidly progressive malignancies; however, this cannot be generalized to all head and neck NMSC, particularly high-risk SCC or tumors in functionally sensitive facial areas. Variations in TTI were observed between regions, likely reflecting differences in local COVID-19 caseloads and healthcare resource allocation.

In our meta-analysis, negative surgical margins were achieved in 88.4% of cases, with no significant difference between the pre-pandemic and pandemic periods. These results are encouraging, as they suggest that short-term oncologic surgical quality, as measured by margin status, was broadly maintained despite the challenges posed by the pandemic. These findings are consistent with national and international benchmarks for NMSC excision. A previous meta-analysis reported pooled clear margin rates of 91.2% (95% confidence interval 87% to 94.6%) for standard surgical excision in head and neck NMSC [31]. Similarly, UK national guidelines from the British Association of Dermatologists consider margin clearance rates above 85% acceptable in routine practice for BCCs [32]. However, margin status alone does not capture the full quality of head and neck NMSC care, particularly reconstructive, functional, aesthetic, and patient-reported outcomes. The high heterogeneity observed in time-to-treatment and histological distribution and the low heterogeneity in reconstruction approaches highlight the variable impact of the pandemic on NMSC care. Regional differences in pandemic severity, healthcare infrastructure, and local policies likely contributed to this variability, underscoring the importance of context-specific data in understanding the broader impact on cancer care. Methodological differences, including study design, follow-up duration, and sample size, may have also influenced these findings.

### 4.2. Comparison with Recent Reports and Clinical Interpretation

The present findings should be interpreted in the context of recent reports describing heterogeneous effects of the COVID-19 pandemic on skin cancer and oncologic care. Large-scale analyses reported substantial reductions in cancer screening, diagnostic procedures, and new cancer diagnoses during the pandemic, raising concern for delayed diagnosis and stage migration [8,9,10]. In skin cancer, however, the reported impact has been less uniform. Some studies described reduced access to care, larger or more advanced cutaneous SCCs, and modified surgical approaches after pandemic-related service disruption [12,33], whereas registry-based or systematic analyses found limited or inconsistent changes in SCC incidence or tumor characteristics [34,35]. In this context, the present review adds a specific head and neck NMSC perspective: despite high heterogeneity in time to treatment initiation, the pooled data did not show a consistent deterioration in histological distribution or surgical margin status, while reconstructive strategy shifted significantly toward primary closure. This suggests that crisis-adapted pathways may have preserved short-term oncologic radicality in selected surgical cohorts, but may still have influenced reconstructive complexity and potentially longer-term aesthetic, functional, and patient-reported outcomes.

### 4.3. Strengths, Limitations, and Future Directions

The main clinical significance of this review is therefore the distinction between preserved short-term oncologic indicators and altered care delivery. The available evidence suggests that crisis-adapted pathways may maintain margin status and histological case mix when prioritization systems are effective. At the same time, the significant shift toward primary closure indicates that reconstructive decision-making may have changed during the pandemic. This information is relevant for post-COVID care because future recovery and contingency pathways should not be evaluated only by treatment volume or margin clearance, but also by reconstructive quality, facial function, aesthetic results, and patient-reported outcomes. Although none of the included studies reported patient-reported outcomes (PROM) or quality of life measures, this remains an important consideration in head and neck NMSC, as recently recommended by The European Academy of Dermatology and Venereology Task Force [36]. Future research should therefore integrate validated QoL instruments alongside oncologic outcomes to better evaluate the long-term impact of crisis-driven treatment strategies [37]. One limitation of this review is the small number of studies meeting the inclusion criteria, which may limit the generalizability of our findings. Furthermore, the reliance on retrospective data increases the risk of bias, as patient selection and reporting inconsistencies may affect study outcomes. In this context, reconstruction outcomes could not be stratified according to histological subtype, so it was not possible to determine whether the observed shift toward primary closure was partially influenced by different proportions of BCC and cutaneous SCC. Finally, variations in healthcare systems and pandemic responses across regions limit the comparability of some findings, suggesting that more standardized, multicenter studies are needed to clarify these preliminary insights.

## 5. Conclusions

This systematic review with exploratory meta-analysis suggests that the COVID-19 pandemic did not uniformly compromise short-term oncologic indicators in surgically treated head and neck NMSC. The histological case mix and surgical margin status remained broadly stable across the included studies. The analysis of reconstruction methods showed a significant shift toward primary closure, with an OR of 1.90 (95% CI: 1.46–2.47; *p* < 0.0001), likely reflecting attempts to reduce operative complexity and resource use. However, this shift raises concerns over potential aesthetic and functional drawbacks, as primary closures may result in less optimal outcomes for complex facial defects. Time to treatment initiation showed substantial heterogeneity, suggesting that the effect of the pandemic varied across healthcare settings according to local organization, pandemic burden, and prioritization pathways. Future studies should investigate the long-term aesthetic, functional, reconstructive, and patient-reported outcomes of crisis-adapted treatment pathways to provide clearer guidance for managing oncologic surgery during crises.

## Figures and Tables

**Table 1:** General characteristics and risk of bias evaluation of included studies.

Author, Year	Nationality	Population	Intervention	Comparison	Outcome	Study Design	NOS	Conclusions
Seretis, 2021 [11]	Greece	Skin cancer	Surgery and reconstruction	pP: May 2019–September 2019 P: May 2020–September 2020	The number and types of skin cancers excised during the study periods, the skin cancer type characteristics, the reconstructive methods used, and the proportion of complete excision margins achieved.	Prospective comparative study	6	COVID-19 created substantial challenges for optimal cancer care, especially for SCC and reconstructive needs in NMSC. Hospitals must restructure to manage skin cancer cases efficiently while safeguarding resources for COVID-19 care.
Cozzi, 2022 [20]	Italy	NMSC	Surgery and reconstruction	pP: December 2019–January 2020 P: December 2020–January 2021	To assess delays in NMSC diagnosis and treatment, tumor size changes, and the prioritization of high-risk cases through “fast-track” referral systems between pre-pandemic and pandemic periods.	Retrospective cohort study	6	Pandemic constraints delayed timely diagnosis and treatment of NMSC. Resources focused on urgent cases, with early-stage cases often delayed. To address backlogs, rapid clearing of waiting lists and efficient use of local anesthesia for less demanding cases are recommended.
Borg, 2022 [13]	UK	Skin cancer	Surgery and reconstruction	pP: January 2018–May 2018 P: January 2020–January 2021	To evaluate the impact of the pandemic on surgical practices for NMSC, including types of reconstruction (primary closure, local flaps), surgical margins, and time to surgery.	Retrospective cohort study	7	Suggested reducing in-hospital visits by maximizing teledermatology for consultations. Emphasized “Green Pathways” and reduced patient exposure in hospital. Advocates for a 31-day target from referral to surgery to align with national guidelines.
Benedetti, 2024 [14]	Italy	NMSC	Surgery and reconstruction	pP: January 2018–December 2019 P: January 2020–December 2021	To investigate changes in the volume of treated H&N NMSC cases, anesthesia type, surgical time, and margin clearance rates in NMSC between pre-pandemic and pandemic periods.	Retrospective cohort study	8	The number of treated NMSC cases did not decrease, but surgical practices shifted to shorter operations and increased use of local anesthesia. Highlights the need for team collaboration to follow guidelines and optimize resources.
Roman, 2024 [21]	Romania	NMSC	Surgery and reconstruction	pP: 2016–2019 P: 2020–2022	To analyze the impact of the pandemic on the incidence of NMSC cases, time to treatment initiation, reconstruction types, and hospital resource allocation.	Retrospective cohort study	8	Observed a reduction of approximately one-third in H&N NMSC cases treated, with increased costs and surgical delays due to COVID-19 precautions. Core treatment standards remained largely stable.

Abbreviations: H&N (Head and Neck); NOS (Newcastle–Ottawa Scale); NMSC (non-melanoma skin cancer); SCC (squamous cell carcinoma); pP (pre-pandemic period); P (pandemic period); UK (United Kingdom).

**Table 2:** Epidemiological data, Staging and Histology.

Author, Year	NOP (pP/P)	Age (pP/P)	Males/Females	NOC (pp/P)	Stage T1 (pP/P)	Stage T2 (pP/P)	Stage T3 (pP/P)	Stage T4 (pP/P)	BCC (pP/P)	SCC (pP/P)	Other (pP/P)
Seretis, 2021 [11]	103/133	73.44/72.09	150/86	125/158	93/73	23/31	6/9	1/0	74/78	29/55	22/25
Cozzi, 2022 [13]	121/93	75.5/76.5	139/75	164/110	NR	NR	NR	NR	119/61	45/49	MD/MD
Borg, 2022 [14]	NR	NR	NR	50/340	NR	NR	NR	NR	32/182	17/145	1/13
Benedetti, 2024 [20]	45/66	77.9/80.39	66/45	63/97	33/50	17/26	9/12	0/0	20/46	42/45	1/6
Roman, 2024 [21]	113/63	72/73	101/75	136/75	85/51	28/16	20/7	3/1	109/57	27/18	MD/MD
Cumulative	382/355	74.20/74.95	456/281	538/780	211/174	68/73	35/28	4/1	354/424	160/312	24/44

Abbreviations: BCC (Basal Cell Carcinoma ); SCC (Squamous Cell Carcinoma ); NOC (Number of Cases); NOP (Number of Patients); NR (Non-Reported); pP (pre-Pandemic); P (Pandemic).

**Table 3:** Surgical and Anesthetic Management and Outcomes of Included Studies.

Author, Year	LA pP/P	GA pP/P	R Primary pP/P	R Flap pP/P	R Skin Graft pP/P	R Matrix pP/P	Margin − pP/P	Margin + pP/P
Seretis, 2021 [11]	NR	NR	33/43	67/37	33/23	NR	85/112	18/21
Cozzi, 2022 [13]	NR	NR	NR	NR	NR	NR	145/99	19/11
Borg, 2022 [14]	43/330	7/10	19/111	26/83	5/24	0/33	49/307	1/33
Benedetti, 2024 [20]	18/55	39/40	5/25	27/44	NR	24/28	51/69	10/25
Roman, 2024 [21]	NR	NR	47/27	87/42	2/6	NR	132/70	4/5

Abbreviations: GA (General Anesthesia); LA (Local Anesthesia); NR (Non Reported); pP (pre-Pandemic); P (Pandemic); R (Reconstruction).

## Data Availability

The data supporting this study’s findings are available on request from the corresponding author.

## Supplementary Materials

The following supporting information can be downloaded at: https://www.mdpi.com/article/10.3390/diseases14070254/s1.
