# Supplementary material for: Oncological Care During a Crisis: A Systematic Review and Meta-Analysis of Non-Melanoma Skin Cancer of the Head and Neck Region in the COVID-19 Pandemic
Source: Diseases. 2026 Jul 14;14(7):254. doi: 10.3390/diseases14070254 (PMC13409594; doi:10.3390/diseases14070254)
Supplement: Supplementary file 1 [file diseases-14-00254-s001.zip › diseases-4328471-supplementary.pdf]

diseases

MDPI

Supplementary Material of “Oncological Care During a Crisis: A Systematic Review and Meta-Analysis of Head and Neck NMSC Management in the COVID-19 Pandemic”

Andrea Frosolini [1], Simone Benedetti [1], Luigi Angelo Vaira [2], Paolo Gennaro [1] and Guido Gabriele [1]

[1] Maxillofacial Surgery Unit, Department of Medical Biotechnology, S. Maria alle Scotte University Hospital of Siena, 53100 Siena, Italy

[2] Maxillofacial Surgery Operative Unit, Department of Medicine, Surgery and Pharmacy, University of Sassari, Sassari, Italy

Search Queries for Systematic Review and Meta-Analysis Runned 4 May 2026 without restriction date.

1. PubMed Search Query (98 Results):

("COVID-19"[MeSH Terms] OR "SARS-CoV-2"[MeSH Terms] OR "pandemic"[Title/Abstract])
AND
("non-melanoma skin cancer"[Title/Abstract] OR "NMSC"[Title/Abstract] OR "Basal cell carcinoma"[Title/Abstract] OR "Squamous cell carcinoma"[Title/Abstract] OR "skin cancer"[Title/Abstract])
AND
("head and neck"[Title/Abstract] OR "scalp"[Title/Abstract] OR "face"[Title/Abstract] OR "neck"[Title/Abstract] OR "craniofacial"[Title/Abstract])
AND
("diagnosis"[Title/Abstract] OR "management"[Title/Abstract] OR "treatment"[Title/Abstract] OR "surgery"[Title/Abstract])

2. Google Scholar Search Query (974 Results):

"COVID-19" AND "Non-melanoma skin cancer" AND "head and neck" AND "treatment" OR "surgery" AND "impact" OR "delayed diagnosis"

3. Scopus Search Query (83 Results):

(("COVID-19" OR "SARS-CoV-2" OR pandemic) AND ("non-melanoma skin cancer" OR NMSC OR "basal cell carcinoma" OR "squamous cell carcinoma") AND ("head and neck" OR scalp OR face OR neck OR craniofacial) AND (treatment OR management OR diagnosis OR surgery OR "therapeutic management") AND (impact OR delay OR "treatment delay" OR "healthcare disruption"))

Data extraction was performed using a standardized data-abstraction form developed for this review. Extracted variables included author, year, country, study design, study period, population, number of patients or lesions, histological subtype, T stage, time to treatment initiation, anesthesia type, reconstructive method, surgical margin status, and risk-of-bias assessment

Supplementary Table S1. Domain-level Newcastle–Ottawa Scale (NOS) assessment of included studies.

Study	Selection	Comparability	Outcome	Total NOS score	Overall risk
Seretis et al., 2021	3/4	1/2	2/3	6/9	Moderate
Cozzi et al., 2022	3/4	1/2	2/3	6/9	Moderate
Borg et al., 2022	3/4	1/2	3/3	7/9	Low
Benedetti et al., 2024	4/4	1/2	3/3	8/9	Low
Roman et al., 2024	4/4	1/2	3/3	8/9	Low
